# Effects of Social Interaction Mechanics in Pervasive Games on the Physical Activity Levels of Older Adults: Quasi-Experimental Study

**DOI:** 10.2196/13962

**Published:** 2019-07-22

**Authors:** Luciano Henrique De Oliveira Santos, Kazuya Okamoto, Silvana Schwerz Funghetto, Adriana Schüler Cavalli, Shusuke Hiragi, Goshiro Yamamoto, Osamu Sugiyama, Carla Denise Castanho, Tomoki Aoyama, Tomohiro Kuroda

**Affiliations:** 1 Graduate School of Informatics Kyoto University Kyoto Japan; 2 Division of Medical Information Technology and Administration Planning Kyoto University Hospital Kyoto Japan; 3 Faculty UnB Ceilândia University of Brasília Brasília Brazil; 4 Escola Superior de Educação Física Federal University of Pelotas Pelotas Brazil; 5 Preemptive Medicine & Lifestyle-Related Disease Research Center Kyoto University Hospital Kyoto Japan; 6 Graduate Program in Informatics University of Brasília Brasília Brazil; 7 Graduate School of Medicine Kyoto University Kyoto Japan

**Keywords:** aged, physical activity, pervasive games, social interaction

## Abstract

**Background:**

The novel genre of pervasive games, which aim to create more fun and engaging experiences by promoting deeper immersion, could be a powerful strategy to stimulate physical activity among older adults. To use these games more effectively, it is necessary to understand how different design elements affect player behavior.

**Objective:**

The aim was to vary a specific design element of pervasive games for older adults, namely social interaction, to test the effect on levels of physical activity.

**Methods:**

Over 4 weeks, two variations of the same pervasive game were compared: social interaction for the test group and no social interaction for the control group. In both versions, players had to walk to physical locations and collect virtual cards, but the social interaction version allowed people to collaborate to obtain more cards. Weekly step counts were used to evaluate the effect on each group, and the number of places visited was used as an indicator of play activity.

**Results:**

A total of 32 participants were recruited (no social interaction=15, social interaction=17); 18 remained until the end of the study (no social interaction=7, social interaction=11). Step counts during the first week were used as the baseline (no social interaction: mean 17,099.4, SE 3906.5; social interaction: mean 17,981.9, SE 2171.1). For the following weeks, changes to individual baseline were as follows for no social interaction (absolute/proportional): 383.8 (SE 563.8)/1.1% (SE 4.3%), 435.9 (SE 574.5)/2.2% (SE 4.6%), and −106.1 (SE 979.9)/−2.6% (SE 8.1%) for weeks 2, 3, and 4, respectively. For social interaction they were 3841.9 (SE 1425.4)/21.7% (SE 5.1%), 2270.6 (SE 947.1)/16.5% (SE 4.4%), and 2443.4 (SE 982.6)/17.9% (SE 4.7%) for weeks 2, 3, and 4, respectively. Analysis of group effect was significant (absolute change: η^2^=.19, *P*=.01; proportional change: η^2^=.27, *P*=.009). Correlation between the proportional change and the play activity was significant (*r*=.34, 95% CI 0.08 to 0.56), whereas for absolute change it was not.

**Conclusions:**

Social interaction design elements of the pervasive game may have some positive effects on the promotion of physical activity, although other factors might also have influenced this effect.

**Trial Registration:**

Japan Medical Association Clinical Trial Registration Number JMA-IIA00314; https://dbcentre3.jmacct.med.or.jp/JMACTR/App/JMACTRS06/JMACTRS06.aspx?seqno=7274 (Archived by WebCite at http://www.webcitation.org/761a6MVAy)

## Introduction

As the proportion of elderly adults increases in populations worldwide, supporting their quality of life has become a pressing social challenge [1]. Many studies have pursued this goal by using electronic games as a novel strategy to address specific issues, such as the rehabilitation of psychomotor functions [2,3], prevention of age-related diseases [4,5], or promotion of active lifestyles [6], all with varied levels of success [7].

Recently, researchers began to explore the new genre of pervasive games [8-12] in this context. A pervasive game is an electronic game that incorporates elements from the real world in its mechanics, blurring the edges of the so-called “magic circle” [13] (ie, the perceived boundaries of the playing space). Among the real-world elements used in such games, two of the most common are physical location and social connections, and both can be beneficial to senior players. When people are invited to walk around and visit places in the real world, they are stimulated to have regular physical activity; when they interact with other people via the social mechanics of the game, it may be possible to reduce or prevent social isolation. Both effects are strongly correlated with higher quality of life among elderly adults and a lower incidence of age-related diseases [14-18].

Few studies have used pervasive games or gamified apps targeting older adults; they usually focus on specific goals, such as cognitive training [19] or the promotion of physical activity using social incentives [20,21]. A successful commercial example that does not target elderly adults specifically that became extremely popular among people of all ages is Pokémon GO [22]. Different studies have analyzed its effects on levels of physical activity and found overall positive results, especially in the first weeks of use [23-25].

However, to effectively use pervasive games to help older players, it is necessary to better understand how different elements of game design can affect their experience. In this work, we investigate whether using social interaction elements in a pervasive game can increase players’ levels of physical activity. Because the main element of mechanics of the game is walking, we assume that if higher levels of physical activity are observed in association with more frequent play activity that also implies that the game experience was more fun and engaging.

## Methods

### Design

The main focus was on evaluating the effects of change in the game design; therefore, we compared two versions of the same pervasive game. The only design element that was changed was social interaction. It was not possible to blindly assign individual participants to different groups because players would be aware of the different interaction options in the game if they interacted with other players. Thus, we compared the versions of the game in two isolated groups in a quasi-experimental study design.

### Participants

Participants were recruited in collaboration with the University of Brasília in Brazil among students who attended classes in a university-run community project that targeted older adults. Students incurred no financial cost to join the project, and no educational background was required, except for being able to read and write. Classes were offered at sites in different regions of the city (more than 30 km apart). Students at different sites did not have contact with one another within the project, but students at the same site attended classes together. Two different sites were chosen to recruit students and form the intervention groups: one group played the version of the game without social interaction and the other group played the version with social interaction. There were no identifiable differences between sites regarding participants’ social, educational, or economic backgrounds.

The inclusion criteria adopted a broader age range of 50 years or older, aiming for middle-aged and older adults, because this research was contextualized as a preventive health intervention and it is expected that experience with games will become increasingly common among older adults in the future. Additional criteria included healthy people with independent ambulation and no cognitive or physical impairment preventing them from understanding the instructions of the game or taking short walks. All participants signed informed consent forms, and the research protocol was approved by the Ethical Committee of the University of Brasília and by the Kyoto University Hospital’s Ethical Committee; both boards report compliance with the Declaration of Helsinki.

### Game

Participants played a pervasive location-based mobile game called *Trilhas* [26,27]. This game has been previously evaluated for its feasibility and adaptability to allow for the testing of different design elements [28].

**Figure 1:**
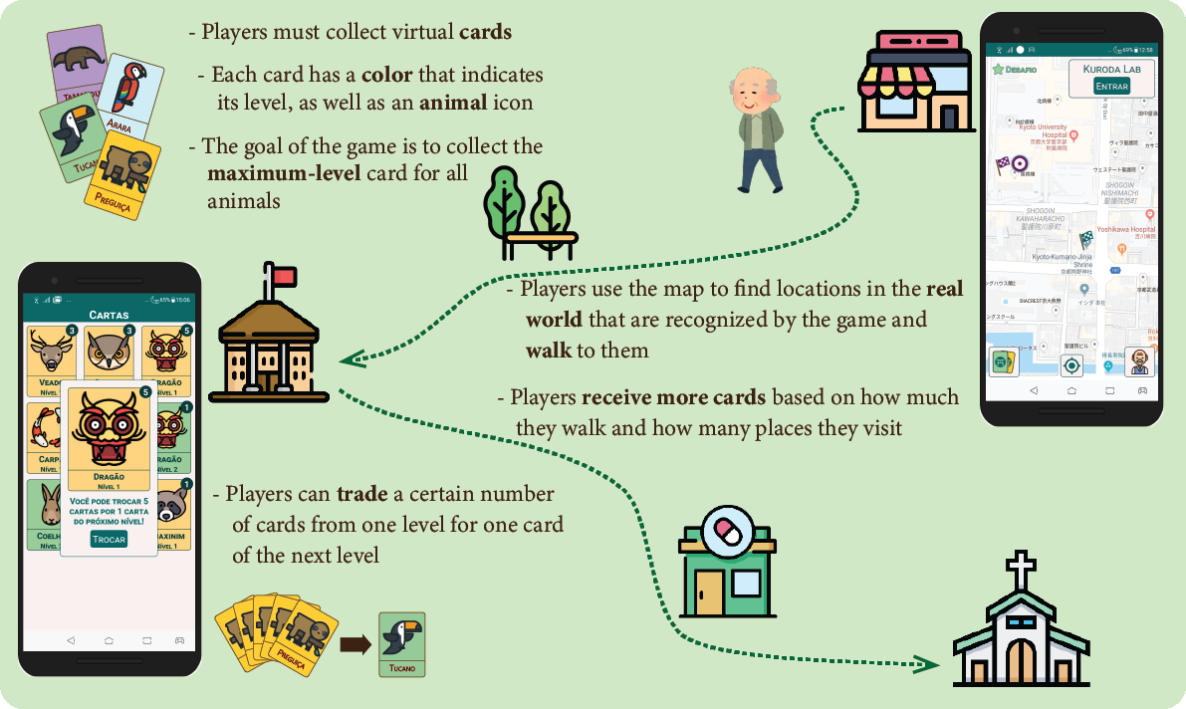
Version of the game without social interaction.

The basic (no social interaction) version of the game (Figure 1) invited players to visit different real-world locations and collect virtual cards. The home screen of the game showed a map that indicated the player’s current location and nearby hotspots (ie, places they can visit). Hotspots were defined using information from Google Maps and included publicly accessible places, such as drugstores, bakeries, coffee shops, churches, and government buildings. Hotspots were spread in a fixed area of the city that included both experiment sites and a range of 20 km, with the distance between hotspots a minimum of 100 m and a maximum of 500 m. If a certain region did not have enough known locations to ensure this distribution, abstract locations were assigned to random physical positions in publicly accessible areas.

After visualizing nearby hotspots, players had to walk toward them. When they were within 50 m of their locations, an “Enter” option appeared on the screen, and players could register their visit. For safety reasons, players were not expected to keep the game screen open while walking, and they were instructed to access the game only when they arrived at their destination. If the game was open and the player’s speed exceeded a certain threshold, the game warned the player not to walk while looking at their mobile phone.

Each day, players received “cards” for the first level proportional to how much they walked (measured by the number of steps) and how many places they visited in previous days. Later, they could trade a certain number of cards from one level for one card of the next level. The goal was to obtain a card of the maximum level for every animal in the game.

**Figure 2:**
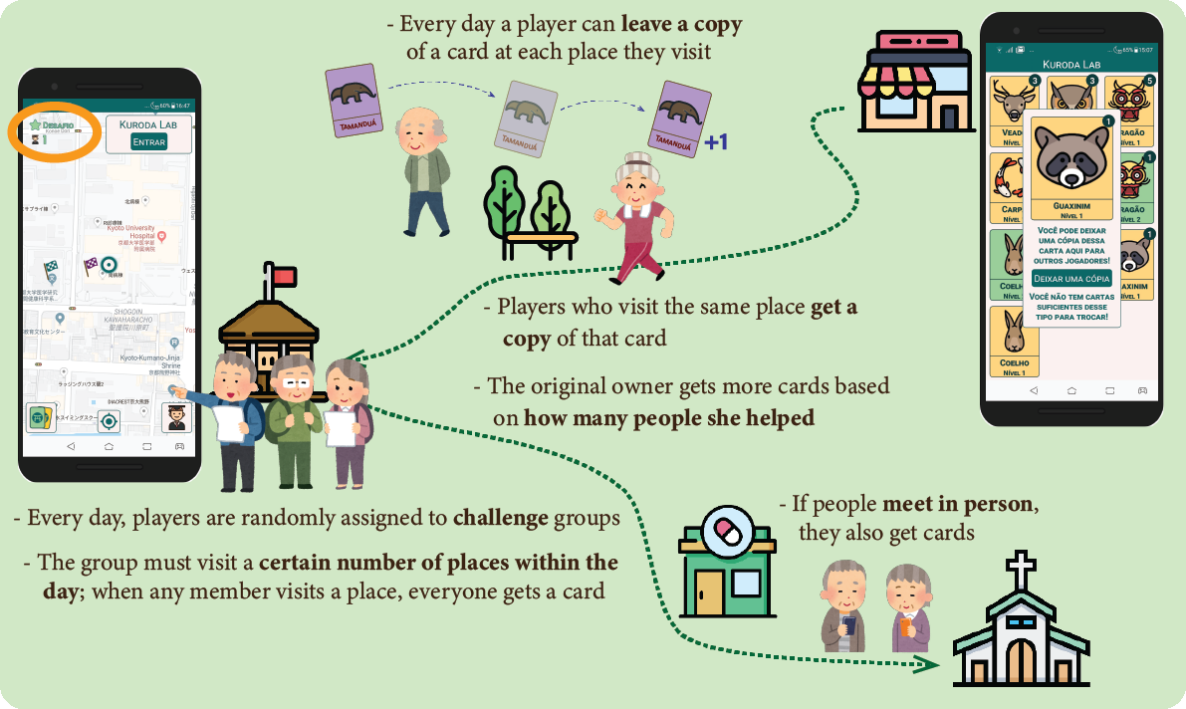
Social interaction version of the game.

In the social interaction version of the game (Figure 2), the following social elements were added to stimulate players to interact and collaborate to obtain more cards:

1. Players could leave copies of their cards on the places they visited, and when other players passed by, they received a copy and the original owner received more cards. When a certain hotspot had a card left on it, its icon exhibited an exclamation mark.
2. Every day, players were randomly assigned to a challenge group, and when a person in the group collected a card, all other members also received a card. Members who contributed to the challenge on that day were shown, whereas players who could not contribute were not shown to avoid “social shaming” (ie, negative reinforcement from other players).
3. When players met in person and scanned each other’s phones, they also received cards.
4. All players could choose a public avatar and nickname and make a short self-introduction. When players received cards from other players’ actions, they had a chance to give them a “like.”

The feasibility study and follow-up evaluations [27,28] suggested that these mechanics allowed players to feel more engaged in playing the game by working together with other people. We hypothesized that this setup would result in a higher positive effect on levels of physical activity.

### Outcome Measures

The main observed outcome was the level of physical activity, measured by the mean number of steps per week over a 4-week period. The step count was measured by the game using a background service that operated whenever the mobile phone was turned on. The software used was Google Android’s Sensor API, which is the same as Google Fit, an app previously shown to have accuracy equivalent to or better than that of wearable devices [29]. During the first week, participants did not play the game, but their step count was still monitored. This monitoring was performed to assess their baseline level of physical activity. After that, they played the game for an additional 3 weeks. To evaluate how much the participants played the game, the weekly mean number of visits to hotspots was also observed. Within a single day, this observation represented the number of unique hotspots visited by the player, whereas within a week, it was the sum of visits each day of the week (ie, the same hotspot was not counted twice for the same day, but it could be counted twice for a week). This measurement was used because players were directed to not keep the game open while walking, so play time was not a good measurement of how much a person played.

As a secondary evaluation, participants were also asked to answer two questionnaires: one assessing their previous experience with games and technology and another evaluating their experience using the game. This second questionnaire was based on the Game Experience Questionnaire [30] and the System Usability Scale [31]. Although these questionnaires are widely used in previous work, they have not been statistically validated yet. For that reason, they served only as complementary information, and the results are reported here for completion. Because the Game Experience Questionnaire was designed to evaluate a broad range of games, including nonelectronic ones, we included only questions related to the gameplay elements present in *Trilhas*. Items used a 5-level Likert scale, indicating the mean agreement level (0=not at all, 4=extremely). They were grouped into categories, with the mean value calculated for each category, as follows:

- Usability:
  - Controls, with items such as “I found the game too complicated to use”
  - Learning curve, with items such as “I could learn how to use the game quickly”
  - Game rules, with items such as “I could understand the game rules”
- Game experience:
  - Theme and visual style, with items such as “I found the game esthetically pleasing”
  - Feeling of immersion, with items such as “I forgot everything about me”
  - Feeling of enjoyment, with items such as “I found the game fun”
  - Feeling of engagement, with items such as “I felt stimulated”
  - Feeling of freedom or ability to explore, with items such as “I felt that I could explore things”
  - Feeling of positive challenge, with items such as “I felt challenged to reach the game’s goals”

The last question of the questionnaire asked participants to freely write comments, criticism, or suggestions. All questionnaires were anonymous.

### Procedures

The sites for social interaction and no social interaction were chosen at random; participants were blinded to group assignment. At the beginning of the study, participants at each site signed the informed consent form and answered the first questionnaire (previous experience with games and technology). Their mobile phone was checked for compatibility, and the game was then installed. Compatible systems included Android-based mobile phones with OS version 5.0 or above with a GPS (Global Positioning System) sensor and an internet connection. Participants who did not have an Android mobile phone or who could not or did not want to use their personal devices were lent a previously prepared one by the researchers.

Participants were told to keep the mobile phone turned on and carry it with them whenever they left their homes throughout the study. There was a follow-up meeting on the same weekday every week, in which researchers were available to clarify any questions or solve technical problems. On the last meeting, after 4 weeks, participants answered the final questionnaire to evaluate their experience while playing.

All questionnaires were administered by researchers, who were available to clarify possible questions about the items.

### Data Analysis

Questionnaire data were consolidated to report percentages in each item, whereas means and standard errors were calculated for demographic data using Google Sheets.

Dropout and step count data were preprocessed using Python (mainly the *pandas* and *matplotlib* packages) to generate graphs and format the data into a suitable format for R. To analyze the effect, we used the change on the number of steps for each week, when compared to the baseline week. This measurement was made for each participant in relation to their own individual baseline, and the proportional change was also calculated (ie, the absolute change divided by the baseline value).

In the statistical model, the change for each week after baseline was considered to be a repeated measure, and an analysis of variance (ANOVA) was performed, with group and week as factors, for participants who remained until the end of the study. Because the experimental setting did not allow for the balancing of data, a type III strategy was used to account for unbalanced data. The relationship between the change in step count and the number of hotspot visits was evaluated using a Pearson correlation coefficient (*r*). This analysis was performed using R.

## Results

### Participants

**Figure 3:**
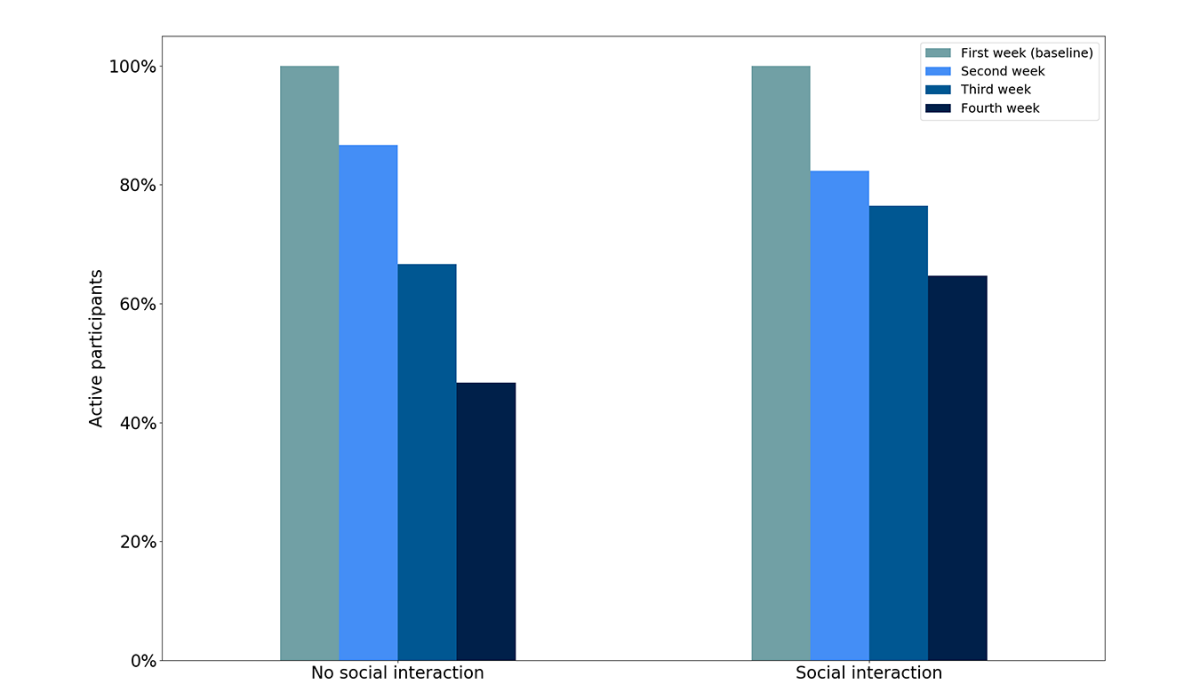
Active participants over time.

The graph in Figure 3 shows the proportion of remaining participants over time. People could drop out at the weekly follow-up meetings, so the data reported here considers participants who stayed for at least the baseline week; otherwise, it was as if they did not join the experiment. The initial number of participants in the no social interaction group was 15 (females: n=11) and 17 (females: n=14) in the social interaction group. At the end of the fourth week, there were 7 (females: n=5) people in the no social interaction group and 11 (females: n=8) people in the social interaction group, indicating dropout rates of 53% and 35%, respectively. Most participants used their own devices. For the no social interaction group, three devices were lent, whereas two were lent for the social interaction group.

One person borrowed a device because they did not own a mobile phone, whereas all others borrowed devices because their own device was incompatible (different OS or unsuitable OS version). Only one person who borrowed a device dropped out (from the no social interaction group), and all other participants who borrowed devices stayed until the end. There was no follow-up to verify the reasons for dropping out, but three participants gave spontaneous reports. One person from the no social interaction group said they did not find the game interesting. One person from the social interaction group said they had back pains that prevented them from walking and another one said they did not have time to play.

Information about participants’ previous experience with technology and games is reported in Table 1. These data include answers collected at the beginning of the study from all participants.

### Main Outcome

Step count data are shown in Table 2 for participants who remained until the end of the experiment. With the absolute change, the analysis of the effect of group as a factor resulted in *P*=.01 (η^2^=.19). No relevant relationship was found with week as a factor (*P*=.65). For proportional change, taking group as a factor resulted in *P*=.009 (η^2^=.27), whereas taking week as a factor resulted in *P*=.54.

For hotspot visits, the group without social interaction had mean 8.4 (SE 2.1) visits in week 2, mean 8.9 (SE 1.7) in week 3, and mean 5.1 (SE 3.0) in week 4. In comparison, the social interaction group had mean 14.2 (SE 1.9) visits in week 2, mean 9.5 (SE 2.0) in week 3, and mean 12.8 (SE 3.4) in week 4.

The correlation analysis between the absolute change in the number of steps and the number of visits resulted in a correlation factor of *r*=.21 (95% CI −.06 to .45). When proportional change was considered, the correlation factor was *r*=.34 (95% CI .08 to .56).

### Game Experience

The scores for the usability and game experience questionnaires are summarized in Table 3. The score of component items could go from 0 to 4; therefore, a value of 2 or greater indicates a positive evaluation. The data included are of only those participants who stayed until the final week.

The questionnaire also included an open-response item in which participants could freely make suggestions and comments.

For the no social interaction group, one participant reported that they often played competitive online games and that *Trilhas* could benefit from a competitive factor. Two participants said they could not play often but wished to have helped more in the research. One participant said the game was boring.

For the social interaction group, six participants said they enjoyed the chance to get more exercise. Three participants said they liked the look of the game, using adjectives such as “cute” and “pleasing.” During the follow-up meetings, five participants commented on the fact they received cards from other participants at specific locations. One participant reported concern about other people knowing their whereabouts.

**Table 1:** Basic data for participants (N=32).

Participant data		Baseline		End of week 4	
		No social interaction	Social interaction	No social interaction	Social interaction
**Demographics**					
	Participants	15	17	7	11
	Sex (female), n (%)	11 (73)	14 (82)	5 (71)	9 (82)
	Age (years), mean (SD)	64.3 (6.0)	61.1 (7.4)	63.9 (5.1)	60.1 (6.0)
	Dropouts, n (%)	8 (53)	6 (35)	—^a^	—
**PC usage frequency, n (%)**					
	Every day	6 (40)	5 (29)	3 (43)	2 (18)
	≥2 times/week	4 (27)	4 (24)	1 (14)	3 (27)
	≤1 time/week	4 (27)	6 (35)	2 (29)	5 (46)
	Never	1 (6)	3 (12)	1 (14)	1 (9)
**PC^b^ experience, n (%)**					
	Able to check email	14 (93)	12 (71)	6 (86)	8 (73)
	Able to do Web searches	14 (93)	12 (71)	6 (86)	8 (73)
	Able to read news online	12 (80)	13 (76)	6 (86)	7 (64)
	Able to use social networks	14 (93)	13 (76)	6 (86)	10 (91)
	Able to install apps	7 (47)	4 (24)	4 (57)	3 (27)
**Mobile phone experience, n (%)**					
	Never used before	1 (7)	1 (6)	1 (14)	1 (9)
	Able to make calls	14 (93)	13 (76)	6 (86)	8 (73)
	Able to check email	13 (87)	13 (76)	6 (86)	9 (82)
	Able to browse the Web	14 (93)	12 (71)	6 (86)	9 (82)
	Able to use social networks	13 (87)	16 (94)	6 (86)	10 (91)
	Able to install apps	6 (40)	8 (47)	2 (29)	6 (55)
**Electronic games play frequency, n (%)**					
	Every day	1 (7)	3 (18)	1 (14)	3 (27)
	≥2 times/week	3 (20)	0 (0)	2 (29)	0 (0)
	≤1 time/week	1 (7)	0 (0)	0 (0)	0 (0)
	Very rarely	1 (7)	2 (12)	1 (14)	1 (9)
	Never plays	9 (60)	12 (71)	3 (43)	7 (64)
**Devices used to play,^c,d,e^ n (%)**					
	Computer	3 (50)	2 (40)	1 (25)	2 (50)
	Mobile phone	3 (50)	4 (80)	2 (50)	4 (100)
	Portable console	0 (0)	2 (40)	0 (0)	2 (50)
	Conventional console	0 (0)	3 (60)	0 (0)	3 (75)
**Play partners,^c,d,e^ n (%)**					
	Plays alone	4 (67)	4 (80)	2 (50)	4 (100)
	Plays with friends	0 (0)	1 (20)	0 (0)	1 (25)
	Plays with adult family members	1 (17)	2 (40)	1 (25)	1 (25)
	Plays with young family members	1 (17)	3 (60)	0 (0)	1 (25)
	Plays with strangers online	1 (17)	0 (0)	0 (0)	0 (0)

^a^Not applicable.

^b^PC: personal computer.

^c^Respondents could indicate more than one item.

^d^Some people reported playing but did not indicate any option on this item.

^e^Percentages are relative to the number of people who reported any play activity.

**Table 2:** Mean number of steps at baseline for each group and mean of the individual variations in subsequent weeks.

Group and value		Baseline, mean (SE)	Mean change at week 2 (SE)	Mean change at week 3 (SE)	Mean change at week 4 (SE)
**No social interaction**					
	Absolute^a^	17,099.4 (3906.5)	383.8 (563.8)	435.9 (574.5)	−106.1 (979.9)
	Proportional (%)^b^	—^c^	1.1 (4.3)	2.2 (4.6)	−2.6 (8.1)
**Social interaction**					
	Absolute^a^	17,981.9 (2171.1)	3841.9 (1425.4)	2270.6 (947.1)	2443.4 (982.6)
	Proportional (%)^b^	—	21.7 (5.1)	16.5 (4.4)	17.9 (4.7)

^a^Absolute values indicate the change in the weekly number of steps compared with the user’s own baseline.

^b^Proportional values indicate the absolute value divided by the user’s own baseline.

^c^Not applicable.

**Table 3:** Results from the usability and game experience questionnaires (N=18).

Category		No social interaction (n=7), mean score	Social interaction (n=11), mean score
**Usability**			
	Controls	2.2	2.7
	Learn curve	2.2	2.6
	Game rules	1.9	2.2
**Game experience**			
	Theme and visual style	2.3	2.5
	Feeling of immersion	2.4	2.2
	Feeling of enjoyment	2.4	2.9
	Feeling of engagement	1.6	2.3
	Feeling of freedom/ability to explore	1.5	2.9
	Feeling of (positive) challenge	1.4	2.1

## Discussion

### Principal Results

At the beginning of the experiment, the majority of participants in both groups had experience with both personal computers and mobile phones. This was also true of those participants who remained until the end of the experiment. When previous experience with games was considered, the majority of participants in both groups reported never playing or playing very rarely. For participants who remained until the end, the ratio of people who played at least once a week increased in both groups. Additionally, participants in both the social interaction and no social interaction groups reported using personal computers and mobile phones to play, but only participants in the no social interaction group used all the devices listed as options. Most people reported playing alone, with the one remaining participant in the social interaction group reporting also playing with friends; the remaining participants in the no social interaction group reported playing with family members and friends.

For the main outcome, a larger positive effect was observed in the social interaction group compared with the no social interaction group. The statistical analysis regarding the absolute change indicated a medium-to-large effect size (η^2^=.19), and the *P* value of .01 indicates a statistically significant difference. There was more variation in the main outcome for the control group, probably due to the higher dropout rate by the end of the third week in that group. A higher number of visits in this group suggests that participants played more, although correlation data were inconclusive: a medium correlation was found for proportional change, but only a small correlation was found for absolute change and that measure was not statistically significant. Therefore, social interaction mechanics may affect player engagement, but other factors may also have influence. Because hotspots are not uniformly spaced, one other possible explanation could be that players tended to visit the same nearby places more often, or visit faraway places only a few times, thus increasing their physical activity to some extent, but not in a linear relationship with the number of visits.

The evaluation of usability and game experience was used as complementary information only. Statistical analysis was not performed because the questionnaires used are not validated. The results indicated an overall positive evaluation for system usability and game experience, as most items had values of 2 or greater. The average evaluation for the social interaction group was higher for all items except immersion, but the difference between groups was small for most items, preventing any solid conclusions. Players in both groups gave positive feedback for the “visual style” of the game and feelings of “immersion” and “enjoyment.” However, there was a larger difference between groups for the categories of “engagement,” “exploration,” and “challenge.” One possible explanation is that the no social interaction group might have had an inferior experience in these categories because the version of the game they played had a subset of the rules of the social interaction group, which could be perceived as less challenging. The usability evaluation for “game rules” was lower in the no social interaction group; however, because the rules in the no social interaction version were a subset of those in the social interaction version, this finding might also be explained by an inferior game experience. Another possible explanation is that some players might not have understood these rules, although players’ comments did not indicate such a case.

In the subjective evaluation considering the open-response comments, players in the social interaction group seemed to have had more fun and felt more engaged in the game, specifically enjoying the card exchange mechanics, although they also felt motivated by the chance of being stimulated to do more exercise. One participant from the no social interaction group complained about the lack of competition, which is a modality of social interaction, leading to the belief that the social factor is relevant for some players. Because these reports were voluntary and many players did not make any comments, it is not possible to generalize these impressions.

### Limitations

There were limitations to this study. The sample size was small, and although the power analysis indicated a medium-to-large effect for relative change and a statistically significant difference for absolute and relative changes, more data could potentially increase the accuracy of these results. Additionally, the dropout rate was high, which could introduce bias toward a positive effect, because the remaining participants might be those who enjoyed the game and were stimulated to continue playing and, potentially, have more physical activity.

The nature of the game and recruitment context made it impossible to use a double-blind design and individually assign participants to groups, which might introduce two biases. First, researchers were aware of group assignments; therefore, they could involuntarily influence participant’s behavior or attitude toward the game during the follow-up meetings. Secondly, even though participants were blind to group assignment, participants in each group had classes together, which might introduce a cohort effect (ie, participants who knew each other and might have a higher tendency to interact using the game and stimulate each other to play). It was also not possible to control for previous experience with technology and games or other possible socioeconomic differences that might have affected the results, although questionnaire data suggest that remaining participants in the no social interaction group had a higher ratio of proficiency to technology, which could have made participants in that group more prone to using the game, in opposition to the observed effect.

The main outcome was measured using mobile phone software. The methodology has been evaluated in previous studies, and the authors of those studies concluded that it is adequate; however, future interventions might test similar settings with a different device, such as external pedometers, and compare the results. In both cases, because the data are not collected in a controlled environment but rather in a user-dependent context, and participant’s adherence to carrying the mobile phone with them was not measured, thus measurements for noncompliant participants are not accurate.

Step counts were observed in a continuous state, considering any daily activity of the participants, and the number of visits to hotspots was used as a proxy measurement to amount of playing because participants are encouraged to only open the game to check in at hotspots and close it between visits. Because step counts for the baseline week were also measured continuously and the analysis considered the observed change, the results are still relevant. Further interventions might also separate in-game counts explicitly and analyze if there is any difference.

Although this study is inserted in the more general field of interventions to improve the quality of life of older adults, it focuses specifically on increasing physical activity based on previous results that showed a strong correlation between these variables. Future interventions could directly focus on these two variables and evaluate their relationship in the context of pervasive games. Also, the questionnaires used to assess usability and game experience were not statistically validated. They could not be used to draw conclusions about the effect and were only complementary information. Using validated metrics would allow for increased data comprehension and the ability to test more hypotheses.

The proposed social interaction mechanics focused mainly on collaboration and virtual interaction. More types of social interaction and different variables can be tested, such as competition, direct (ie, in-person) interaction, group dynamics, and interaction with family and friends, among others.

### Comparison With Prior Work

Although games have been used many times previously to promote the well-being of elderly people, there is usually an excessive focus on health benefits, with little attention given to aspects such as motivations to play and overall game experience. Recently, new research has emerged [32,33] that analyzes in greater depth the experience of elderly people in play based on the principle that games, even serious games, should ﬁrst be fun because the health benefits come later as a natural consequence [34]. This notion aligns with the idea that a deep and meaningful connection with play and fun is an inherent part of human nature [35], and elderly people are no exception. In that respect, few studies have attempted to clarify elderly players’ needs and motivations and investigated possible challenges in designing for older audiences, listing common physical and cognitive limitations that should be taken into consideration [36-40]. Other studies have attempted to identify the preferences of elderly people regarding the content or genre of the games [41-44]. In this study, we evaluated social interaction as a design element in the context of pervasive games, which is a new kind of game that is only now being explored. This study was limited and focused on a specific metric, namely physical activity, which was used as a proxy, but the results suggest that this topic should be further investigated, with the consideration of additional variables related to game experience.

Regarding interventions that promoted walking in general [45], the most effective studies that were analyzed achieved a net increase of 30 to 60 minutes of walking per week. Considering the conversion criteria used in that systematic review, this is equivalent to 3000 to 6000 steps. This study achieved those numbers for the social interaction group in the first week, but those results were not sustained over time. More investigation is necessary to explore how player engagement could be maintained for longer periods.

### Conclusions

In this work, we investigated whether the new genre of pervasive games could be used to increase physical activity of older adults. Our results indicated that a pervasive game using social interaction had a greater positive effect on levels of physical activity than the same game without social interaction. This study was limited; these results are promising but not conclusive. In future interventions, other types of social interaction or design elements should be evaluated, and additional variables considered, such as indicators of physical and psychological health among others.

## Abbreviations

ANOVA: analysis of variance
GPS: Global Positioning System
